# PlantMW*pI*DB: a database for the molecular weight and isoelectric points of the plant proteomes

**DOI:** 10.1038/s41598-022-11077-z

**Published:** 2022-05-06

**Authors:** Tapan Kumar Mohanta, Muhammad Shahzad Kamran, Muhammad Omar, Waheed Anwar, Gyu Sang Choi

**Affiliations:** 1 Natural and Medical Sciences Research Center, University of Nizwa, Nizwa, 616 Oman; 2 Department of Computer Science and IT, The Islamia University of Bahawalpur, Bahawalpur, Pakistan; 3 Department of Data Science, Faculty of Computing, The Islamia University of Bahawalpur, Bahawalpur, Pakistan; 4 Department of Information and Communication Engineering, Yeungnam University, 214-1, Gyeongsan-si, 712-749 South Korea

**Keywords:** Plant biotechnology, Proteins, Computational biology and bioinformatics, Plant sciences

## Abstract

The molecular weight and isoelectric point of the proteins are very important parameters that control their subcellular localization and subsequent function. Although the genome sequence data of the plant kingdom improved enormously, the proteomic details have been poorly elaborated. Therefore, we have calculated the molecular weight and isoelectric point of the plant proteins and reported them in this database. A database, PlantMWpIDB, containing protein data from 342 plant proteomes was created to provide information on plant proteomes for hypothesis formulation in basic research and for biotechnological applications. The Molecular weight and isoelectric point (*pI*) are important molecular parameters of proteins that are useful when conducting protein studies involving 2D gel electrophoresis, liquid chromatography-mass spectrometry, and X-ray protein crystallography. PlantMWpIDB provides an easy-to-use and efficient interface for search options and generates a summary of basic protein parameters. The database represents a virtual 2D proteome map of plants, and the molecular weight and *pI* of a protein can be obtained by searching on the name of a protein, a keyword, or by a list of accession numbers. The PlantMWpIDB database also allows one to query protein sequences. The database can be found in the following link https://plantmwpidb.com/. The individual 2D virtual proteome map of the plant kingdom will enable us to understand the proteome diversity between different species. Further, the molecular weight and isoelectric point of individual proteins can enable us to understand their functional significance in different species.

## Introduction

Each and every molecule in a cell has its own special characteristics, including the individual proteins that comprise the proteome of an organism^1,2^. Proteomes comprise all of the translated products of nucleotide sequences contained in messenger RNA (mRNA)^3–5^. The total mRNA of an organism encodes a wide array of proteins that vary in cellular function and homeostasis^6–8^. These proteins have diverse molecular weights and isoelectric points (*pIs*)^1,2^. Post-translational modifications that occur can alter the function of a protein and contribute to the ability to target the location of a protein to a specific subcellular compartment^9–11^. The shape, size, solubility, and *pI* of a protein determine its ability to move across different cellular compartments and also determine their function^12–17^. Plant cells contain a vast array of proteins with different molecular weight and *pI*^1^. The *pI* indicates the *pH* at which the net charge of a protein is zero^1^. The dissociation constant (*pK*_*a*_) of a polypeptide is determined by the presence of seven different charged amino acids; arginine, aspartate, cysteine, glutamate, histidine, tyrosine, and lysine^18–20^. The N-terminal NH_2_- and C-terminal COOH-group of a protein also influences the charge of a polypeptide^21–25^. Post-translational modifications, protein–protein interactions, dipole interactions, and other biochemical factors also influence the *pI* of a protein^26–30^. Molecular weight and *pI* are used to determine the position of a protein sequence in a proteome map and provide useful information to bioinformatics and genome scientists seeking to understand the molecular basis of subcellular localization and function^31,32^. Several attempts have been made to create a database of experimentally validated proteins^33–38^. It is difficult, however, to experimentally validate the *pI* and molecular weight of each and every individual protein in a proteome. Previous databases have contained experimentally validated *pI* data on a maximum of five thousand proteins, which is a relatively low number compared to the number of proteins present in the whole proteome of a species^39,40^. Therefore, we constructed a database containing the *pI* and molecular weight representative of the entire plant kingdom by including protein sequences from the whole proteome of 342 plant species. The PlantMWpIDB database comprises 6.115 million proteins sequences present in the plant kingdom. PlantMWpIDB has a search engine that allows one to explore a virtual 2D map of the global, plant proteome, and search by protein name, keyword, accession number, and protein sequence.

## Construction and content

PlantMWpIDB is a novel database containing proteomic information on 342 plant species. The study contains a total of 13.82 million protein sequences. The molecular weight and isoelectric point of plant proteins were calculated using the Linux-based isoelectric point calculator^41^ and the result obtained from the analysis was used to construct the database. PlantMWpIDB provides a user-friendly interface and display of information. It provides a user the ability to search for information about a specific protein of a species and provides a summary of information and statistics on plant proteomes.

Annotated protein sequences of plant species were downloaded from the National Center for Biotechnology Information (NCBI), Phytozome, and Uniprot in fasta/fna format. The downloaded protein files were used to calculate the predicted molecular weight and isoelectric (p*I*) point of all the proteins. The latter was determined using a protein isoelectric point calculator (http://isoelectric.org/) (IPC Python) within a Linux-based platform^41^. The IPC program provided the molecular weight and isoelectric point of the individual protein sequences. The results were subsequently processed using Microsoft Excel 2016.

The database provides three types of searching and browsing information:

- Scatter plot image of the proteome of an individual species, which is referred to as a virtual 2-D map of the proteome of a species.
- Text information about each species, including the accession number of each of the proteins, protein names, molecular weight (kDa), and isoelectric point (*pI*).
- Protein sequences.

There are three types of entities in the database:

- “species”, for storing plant species protein information.
- “species map”, for storing a virtual 2D proteome map of each species.
- “protein sequence”, for storing proteins sequences of each species.

**Figure 1:**
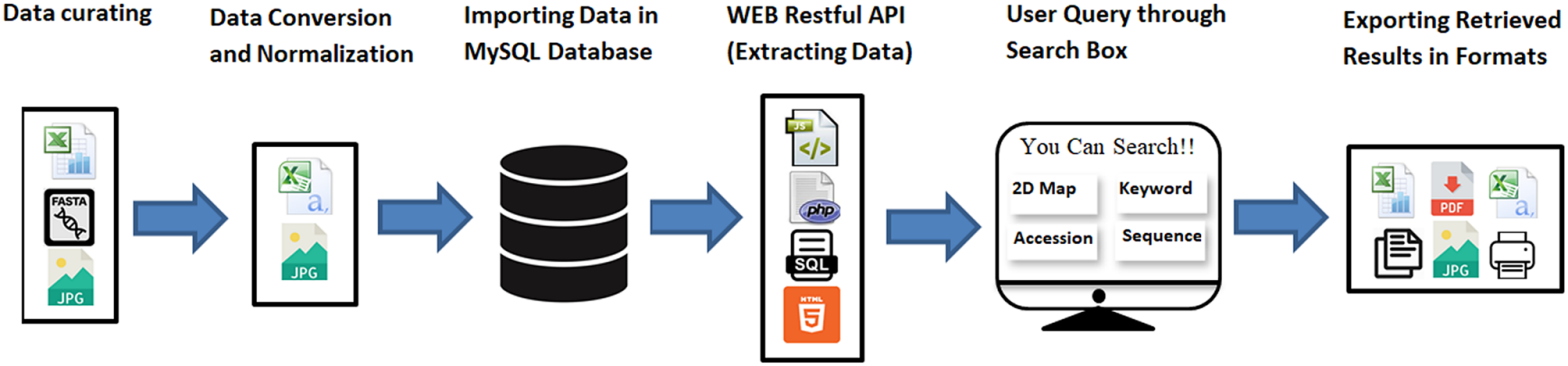
Flowchart depicting the design of PlantMWpIDB of plant proteomes.

**Figure 2:**
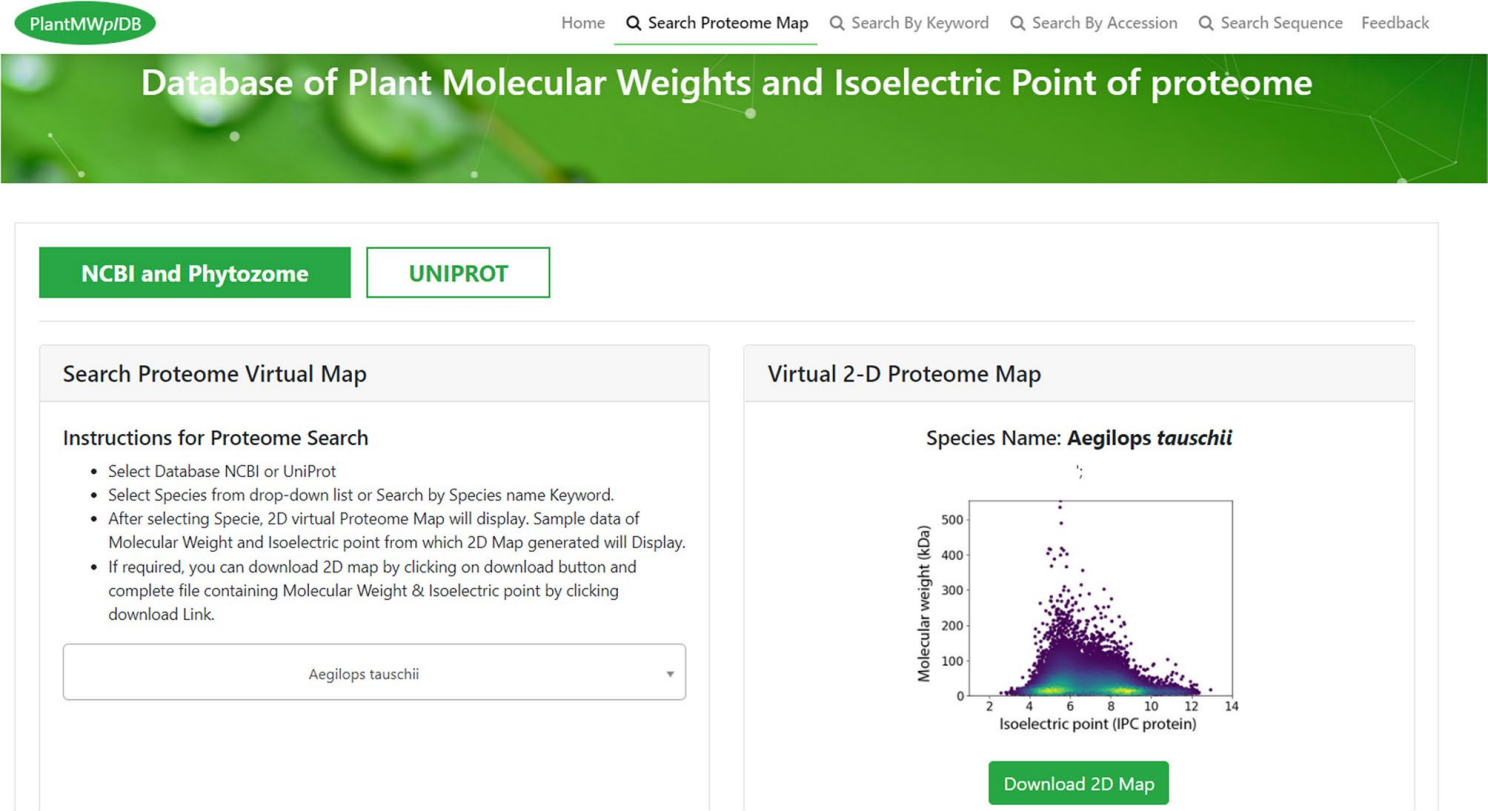
Pictorial presentation of PlantMWpIDB interface. The database shows the search box for the individual species to find the molecular weight and isoelectric point of the plant protein. The user can use the search box using keyword/protein name in any specific species to search the molecular weight and isoelectric point of the protein associated with the key words.

The detailed pipeline used for the construction of PlantMWpIDB is presented in Fig. 1. Users can access the sequence, molecular mass, and *pI* of a specific protein by providing the accession number of the protein. Users can also use keywords or protein names to find the molecular weight and *pI* of a protein of the respective species (Fig. 2). Users can also extract a virtual 2D proteome map of an individual species by browsing the name of a species in the module panel (Fig. 2). The virtual 2D map of a plant proteome represents an image of a virtual two-dimensional polyacrylamide gel electrophoresis (2D-PAGE) gel.

## Languages and tools

User interfaces were developed to provide easy-to-use and efficient access to data. The static interfaces were developed using Hypertext markup language (HTML) and CSS. Restful web API (JSON) and JQuery were used to provide efficient access to web pages. Java Script was used to avoid repeated reloading of pages when a user makes a query using text boxes, thus, improving the efficiency of the interface. Server-side programming language (Php) and query processing language (SQL) was used for developing access to the data within the database. Tables are used to display the data, and each table provides options to copy and save the retrieved information, with the ability to export the information in a variety of file types, including Excel, csv, pdf. Print options using JQuery are also available. The design structure of the PlantMWpIDB, web-based database is presented in Fig. 1.

## Construction of modules

PlantMWpIDB has five main modules:

- search virtual 2D map of the plant proteome.
- search by protein name keyword(s).
- search by accession number(s).
- search by protein sequence.
- Summary statistics.

Using the module, “search virtual 2D map of the plant proteome”, allows one to find the virtual 2D map which is constructed using a scatter plot program and is based on Molecular mass (KDa) and Isoelectric point (*pI*) protein data of each species. The summary statistics contain the proteomic details of proteomes downloaded from NCBI, Phytozome, and Uniprot.

The construction of the two modules—“Search Proteins by Protein Name Keyword”, and by “Accession Number”, were completed in two steps:

- **Preprocessing of the data:** The following protein data were collected from each species and placed in a Microsoft excel file which was generated through IPC software^41^; accession number, protein name, molecular mass, and isoelectric point of each species. The excel files were then converted to comma-separated files (csv) to import the data into MySQL.
- **Database Design:** The database was designed in MySQL Server. The structure of the database tables is presented in Supplementary Table 1. After creating database tables, for all of the 342 database tables, they were imported into the MySQL Server.

The construction of the “search protein sequence” was completed in two steps:

- **Preprocessing of the data:** For the development of this module, Fasta files were converted to a comma-separated format (csv) using a python language script and then imported into the MySQL Server. The csv files were then compressed for efficient memory use.
- **Database Design:** Tables were created for the database for the protein sequences of each of the plant species. After creating the database tables for all species, they were imported using a command prompt. The database contains protein sequence tables for each of the 342 plant species.
- Proteome statistics are efficiently calculated using SQL queries. The summary statistics table of all the proteomes has a sorting option for each column that further facilitates data analysis.

## Results

The module provides users an overview of the overall statistics of the database. The general statistics provided for the proteome of each species include:

- Sequence count.
- Average molecular weight.
- Average *pI*.
- Number of acidic *pI* proteins.
- Percentage of acidic *pI* proteins.
- Number of basic *pI* proteins.
- Percentage of basic *pI* proteins.

The overall statistics of the PlantMWpIDB database (https://plantmwpidb.com/) are provided in Table 1.

**Table 1:** Statistics of plant proteomes downloaded from different sources.

Proteomic parameters	Proteomes from NCBI and phytozome	Proteomes from uniprot
Total number of proteome	147	195
Total number of protein sequences	6,115,918	7,713,142
Average molecular weight of plant proteome	1,965,567 kDa	1,701,111 kDa
Average molecular weight of plant protein	47 kDa	44 kDa
Average *pI*	6.84	6.88
Number of acidic *pI* proteins	3,427,218	4,212,269
Percentage of acidic *pI* proteins	56	54
Number of basic *pI* proteins	2,674,959	3,484,682
Percentage of basic *pI* proteins	43	45
Total number of neutral *pI* proteins	13,741	16,191
Percentage of neutral *pI* proteins	0.21	0.2

The main purpose of PlantMWpIDB (https://plantmwpidb.com/) is to facilitate the ability to obtain information on a specific protein in a specific species or groups of species. Different methods can be used to extract the proteomic data on a protein within an individual species.

- Searching a Virtual 2D Map of Plant Proteomes
- This module provides the facility to search or browse a virtual 2D map of the proteome of a given plant species. The interface is shown in Fig. 2. The interface has two options (1) *pI* and molecular weight of proteins downloaded from NCBI and phytozome and (2) *pI* and molecular weight proteins downloaded from Uniprot.
- This interface has a list box with species names, along with a keyword search option, instructions for using the module, and a map window for displaying the virtual 2D map of the proteome of a selected species. By default, it will display the virtual 2D map of the proteome of the first species on the list.
- Users can download the raw data of *pI* and molecular weight of individual species. Also, users can download the 2D proteome map of a selected species.

### Search proteins by protein name or keyword

This module provides the user with the ability to search for information on a protein using an accession number, protein name, molecular weight, or isoelectric point (*pI*) within a species or by a keyword related to the protein name (Fig. 2). The user also has the ability to save or print the information once it has been retrieved from the database. The interface has a list box of species names and a keyword search option. A text box is provided for entering a protein name or keyword. Instructions for using the module are also provided. The interface also includes a window for displaying search results for a given species. By default, it will display two results within the first species on the list for entered keywords. The interface has options for copying or printing the retrieved results. Alternatively, the retrieved results can be saved in several file formats, including an Excel file, pdf, or csv file. This interface is presented in Fig. 2.

### Sub-search option on retrieved search results

The sub-search option queries the table that is formed from retrieved data. It queries all the columns of data within the retrieved table. For example, in searching for a protein by name, a user can search a keyword of a protein name from the interface. The retrieved results of the search will be presented in a table. Users can then use the sub-search option to locate a specific entry or entries in the retrieved results.

### Sorting options on each column

Users also have the option of sorting any column within the retrieved results table (see point 2).

### Search proteins by accession number

This provides the ability to search the database using the accession number, protein name, molecular mass, or isoelectric point (*pI*) within a specific species by using a single accession number or a list of accession numbers. The user can also save the retrieved information for subsequent use. The interface has a list box of species names, along with a keyword search option. A text box for entering a single or list of an accession number(s), and instructions for using this module are also provided and there is a window that displays the search results within a selected species. By default, it will display two results within the first two accession numbers of the first species in the list. The module has options that allow the user to copy, print, or export the retrieved results as an Excel file, pdf, or csv file.

### Search by protein sequence

This module provides the ability to search the database using a protein sequence within a species using a list of accession numbers provided by the user. The interface has a list box of species names with a protein name or keyword search option. A text box is provided for entering a list of accession numbers, and instructions for using this module are also provided. The interface also includes a window for displaying sequences within a selected species name. By default, it will display two results within the first two accession numbers of the first species in the list. As with other modules, the interface provides option to option copy, print, or save the retrieved data as an Excel file, pdf, or csv file.

## Error messages within the different modules

All of the modules will provide an alert (error message) if incomplete or incorrect data are entered into the search options as follows:

- If the user clicks on a button with an empty search box field and list box, the system will display the message, “Please select options and enter search data in the search box”, via an alert box.
- If the user clicks on a button with an empty search box field and selects the list box, the system will display this message, “Please enter search data in the search box”, via an alert box.
- If the user enters a keyword in the search box field with length of less than four characters, the system will display the message, “Please enter valid Accession number of at least four characters or comma separated list of accession numbers in the search box”, via an alert box.
- If no results are found in the database, the system will display the message, “Sorry! Matching records are not found in the database”, via an alert box.

## Discussion

Several databases are available on the web for use by genomic researchers that provide different types of information on either a small or large scale. Proteome-*pI* and Proteome-pI 2.0 is a database having data of isoelectric point of several proteomes^40,42^. Recently, Kozlowski (2022) reported the Proteome pI-2.0: proteome isoelectric point database where the author reported the molecular weight, isoelectric point, and enzymatic digestion details of 61.329 million protein sequences from 20,115 proteomes^42^. They were from eukaryote, bacteria, archaea, and viruses^42^. From the mentioned eukaryotic species, Kozlowski (2022) reported the molecular weight and isoelectric point of more than 250 plant species. However, a lack of proper classification makes it difficult to find out the plants or animal species from a large number of species^42^. Therefore, we constructed PlantMWpIDB and reported here. Our study provided the proteomic data of 342 plant species from the proteomic sequences of NCBI, Phytozome, and Uniprot. The plant proteomic database (PPDB) provides proteomic data on *Arabidopsis* and maize^4^. A database on moonlighting plant proteins (PlantMP) contains protein functions searchable by UniProt IDs and names, canonical and moonlighting functions, or gene ontology numbers^43^. The *Arabidopsis* Nucleolar Protein Database (AtNoPdb) provides information on 217 proteins found in *Arabidopsis*^44^. In general, these databases provide information on a limited number of plant species and/or a limited number of proteins in a sporadic manner.

Although, Proteome-pI 2.0 provided the proteomic data of more than 250 plant species, a lack of specific classification making it difficult to identify which of the data is plant, animal, or fungi species. Therefore, we have constructed PlantMWpIDB based on the 342 species of the plant kingdom to provide information on the molecular weight and isoelectric point of 13.82 million protein sequences. In addition, the Proteome-pI 2.0 do not have any option to search for the molecular weight and isoelectric point of any particular protein using the “keyword”, “accession number”, or protein sequence^42^. For this, a user need to download whole data file to find the molecular weight and isoelectric point of a single protein. Also, Proteome-pI 2.0 has provided the isoelectric point of protein sequences using 21 different parameters including Bjellqvist, DTASelect, Dawson,EMBOSS, Grimsley, IPC2_peptide, IPC2_protein, IPC_peptide, IPC_protein, Lehninger, Nozaki, Patrickios, ProMoST, Rodwell, Sillero, Solomon, Thurlkill, Toseland, Wikipedia, IPC2.protein.svr19, and IPC2.peptide.svr19^42^. It will be difficult for the user to decide which isoelectric point is correct for a particular protein sequence. However, our study revealed isoelectric point obtained using IPC_protein best suit with the native isoelectric point of the protein. Therefore, we have constructed our database using the feature of IPC_protein.

The isoelectric point of a protein represents the *pH* at which the net charge of the protein is zero, and represents an important analytical and molecular parameter^1^. The *pI* of a protein is often used in biochemistry to determine differential expression of a protein based on 2D-PAGE gel electrophoresis, x-ray protein crystallography, and capillary isoelectric focusing^45–47^. The global virtual 2D map of the plant kingdom exhibited a trimodal distribution within the global proteome, with acidic *pI* proteins dominating over the basic *pI* proteins^1^. This suggests that the *pI* of the majority of plant proteins are close to the physiological *pH* of a cell. In our previous study^1^, we reported that the molecular weight in the global plant proteome ranged from 0.54 to 2236.8 kDa and the isoelectric point ranged from 1.99 to 13.96^1^. The acidic *pI* proteins of monocot plants were closely correlated with the acidic *pI* proteins of bryophytes, while they were distantly related to algae and eudicot plants^1^. The amino acid composition of the global plant proteome was observed to be lineage specific^1^. The amino acid composition of algae, monocot, and eudicot proteome form independent clusters. Leu, Ser, Ile, Lys, and Gln are amino acids that are highly abundant in the plant proteome, while Tyr, Trp, Cys, His, and Met are low abundant amino acids^1^.

### Future perspectives

The PlantMWpIDB database contains the molecular  weight and isoelectric point of the plant proteomes of 342 species, including algae, bryophytes, pteridophytes, gymnosperms, monocots, and eudicots. Future versions of the PlantMWpIDB database will include protein molecular modeling module to decipher the 3-D structure of each protein. Future versions will also include target site prediction for metacaspases, palmitoylation, myristiylation, and methylation for each protein. Collectively, this additional information will provide important information to researchers investigating protein modification, function, structure, and evolution.

## Conclusion

PlantMWpIDB provides researchers with the ability to retrieve information on the molecular mass and pI of proteins within the proteome of 342 plant species, ranging from algae to eudicots. PlantMWpIDB is the most comprehensive database available on plant proteomes and contains several modules for searching, retrieving, and saving data. Future versions of PlantMWpIDB will make the database even more powerful for obtaining information on the proteome of the entire plant kingdom.

## Supplementary Information

Supplementary Information.
