# Supplementary material for: PlantMWpIDB: a database for the molecular weight and isoelectric points of the plant proteomes
Source: Sci Rep. 2022 May 6;12:7421. doi: 10.1038/s41598-022-11077-z (PMC9076895; doi:10.1038/s41598-022-11077-z)
Supplement: Supplementary file 1 — Supplementary Information. [file 41598_2022_11077_MOESM1_ESM.docx]

| - Sr# | - Attribute Name | - Data Type | - Size | - Constraints |
| --- | --- | --- | --- | --- |
| 1 | - Accession_Number | - Varchar | - 14 | - Primary Key |
| - 2 | - Protein_Name | - Varchar | - 127 | - Not Null |
| - 3 | - Mol_Weight | - Decimal | - 8,2* | - Not Null |
| - 4 | - Mol_Weight_kDa | - Decimal | - 8,5** | - Not Null |
| - 5 | - pI | - Decimal | - 5,3* | - Not Null |

Supplementary Table 1. Structure of database tables for storing species details.
